# Variations of pulse pressure and central venous pressure may predict fluid responsiveness in mechanically ventilated patients during lung recruitment manoeuvre: an ancillary study

**DOI:** 10.1186/s12871-022-01815-1

**Published:** 2022-08-23

**Authors:** Olivier Desebbe, Whitney Mondor, Laurent Gergele, Darren Raphael, Sylvain Vallier

**Affiliations:** 1 Department of Anesthesiology and Intensive Care, Ramsay Sante Sauvegarde Clinic, Lyon, France; 2 Department of Biosciences, Claude Bernard University, Lyon, France; 3 Department of Anesthesiology and Intensive Care, Ramsay Sante HPL Clinic, Saint-Etienne, France; 4 Department of Anesthesiology & Perioperative Care, University of California, Irvine, USA; 5 Department of Anesthesiology and Intensive Care, Elsan Alpes-Belledonne Clinic, Grenoble, France

**Keywords:** Pulse pressure, Central venous pressure, Lung recruitment manoeuvre, Fluid responsiveness

## Abstract

**Background:**

Maintaining a constant driving pressure during a prolonged sigh breath lung recruitment manoeuvre (LRM) from 20 to 45 cmH_2_0 peak inspiratory pressure in mechanically ventilated patients has been shown to be a functional test to predict fluid responsiveness (FR) when using a linear regression model of hemodynamic parameters, such as central venous pressure (CVP) and pulse pressure (PP). However, two important limitations have been raised, the use of high ventilation pressures and a regression slope calculation that is difficult to apply at bedside. This ancillary study aimed to reanalyse absolute variations of CVP (ΔCVP) and PP (ΔPP) values at lower stages of the LRM, (40, 35, and 30 cm H_2_0 of peak inspiratory pressure) for their ability to predict fluid responsiveness.

**Methods:**

Retrospective analysis of a prospective study data set in 18 mechanically ventilated patients, in an intensive care unit. CVP, systemic arterial pressure parameters and stroke volume (SV) were recorded during prolonged LRM followed by a 500 mL crystalloid volume expansion. Patients were considered as fluid responders if SV increased more than 10%. Receiver-operating curves (ROC) analysis with the corresponding grey zone approach were performed.

**Results:**

Areas under the ROC to predict fluid responsiveness for ΔCVP and ΔPP were not different between the successive stepwise increase of inspiratory pressures [0.88 and 0.89 for ΔCVP at 45 and 30 cm H_2_0 (*P* = 0.89), respectively, and 0.92 and 0.95 for ΔPP at 45 and 30 cm H_2_0, respectively (*P* = 0.51)]. Using a maximum of 30 cmH_2_O inspiratory pressure during the LRM, ΔCVP and ΔPP had a threshold value to predict fluid responsiveness of 2 mmHg and 4 mmHg, with sensitivities of 89% and 89% and specificities of 67% and 89%, respectively. Combining ΔPP and ΔCVP decreased the proportion of the patients in the grey zone from 28 to 11% and showed a sensitivity of 88% and a specificity of 83%.

**Conclusions:**

A stepwise PEEP elevation recruitment manoeuvre of up to 30 cm H_2_0 may predict fluid responsiveness as well as 45 cm H_2_0. The combination of ΔPP and ΔCVP optimizes the categorization of responder and non-responder patients.

**Supplementary Information:**

The online version contains supplementary material available at 10.1186/s12871-022-01815-1.

## Introduction

Fluid therapy is a key component of cardiac output optimization [1]. However, analysis of clinical studies on the hemodynamic effects of volume expansion (VE) reveals that only 50% of patients respond to VE with a significant increase in stroke volume (SV) [2]. This underscores the utility of hemodynamic manoeuvre tests such as passive leg raising [3] or dynamic variations of intrathoracic pressure [2] to avoid unnecessary or even deleterious fluid intake in "nonresponder" patients, in whom inotropic and/or vasoactive agents should be used preferentially to improve hemodynamic status [4].

Lung recruitment maneuvers (LRM) have been proposed in mechanically ventilated patients to optimize oxygen delivery by increasing gas exchange and to decrease pulmonary complications in both the perioperative setting and in acute respiratory distress syndrome patients [5, 6]. LRM can be performed using a stepwise increase in PEEP and airway inspiratory pressure with a constant driving pressure [7]. We recently studied multiple parameters during a six step PEEP elevation recruitment manoeuvre in ICU patients [8]. Areas under the receiver-operating curves (AUC) showed that the best parameters for fluid responsiveness prediction were the slope angle for pulse pressure (AUC = 0.93 95% CI 0.78 to 1.00 sensitivity 100% specificity 89%) and the slope angle for central venous pressure (AUC = 0.90 95% CI 0.76 to 1.00). By combining sensitivity of PP angle and specificity of CVP angle, fluid responsiveness prediction could be obtained with 100% sensitivity and 100% specificity (AUC = 0.96 95% CI 0.90 to 1.00). However, reviewer comments appropriately raised the issues of using a slope calculation that is difficult to apply at bedside and the use of high inspiratory pressures (from 20 to 45 cm H_2_0) during the LRM which may have deleterious physiological impacts [9].

This ancillary study aimed to analyse absolute variations of CVP (ΔCVP) and PP (ΔPP) values at lower stages of the step PEEP LRM (40, 35, and 30 cm H_2_0 of peak inspiratory pressure) for their ability to predict fluid responsiveness solely and by combining both parameters.

## Material and methods

Respiratory and hemodynamic data from 18 mechanically ventilated and sedated ICU patients included in the previous prospective study were analysed [9]. The initial study protocol was approved by the hospital’s ethics committee (Ethics Committee, Department of Anaesthesiology, Saint-Etienne University Central Hospital, institutional Review Board IORG0007394, Protocol number IRBN902018/CHUSTE). Patient characteristics and hemodynamic monitoring have been described previously [9]. Briefly, all patients were monitored with a central venous pressure monitoring and an invasive arterial pulse contour analysis (PICCO system, Pulsion Medical Systems SE, Feldkirchen, Germany) for continuous cardiac output monitoring and transpulmonary thermodilution measurement before the LRM and after the VE. The use of protective mechanical ventilation and need for VE were deemed necessary. Sedation was maintained with propofol and/or midazolam (in combination with sufentanil or remifentanil).

### Lung recruitment manoeuvre and volume expansion

The LRM consisted of a 30 s stepwise increase in PEEP from 5 to 30 cm H_2_O while maintaining a constant driving pressure of 15 cmH_2_O (Supplementary Figure S1) [5, 7]. PEEP was then symmetrically decreased from 30 to 5 cmH_2_O. A video of the hemodynamic monitoring was recorded during the LRM, with the clinician announcing the time and pressure level for each step. Hemodynamic values were later documented by pausing the video during the two last seconds of each LRM step as announced by the physician. ΔPP and ΔCVP were respectively calculated as the difference of the average PP and CVP value during the LRM step (mean value during the respiratory cycle) and the baseline (mean value during the baseline respiratory cycle). Next, a volume expansion (VE) of crystalloid (Plasmalyte, Baxter®, Belgium) 500 mL over 10 min was performed, as prescribed by the current standard care protocol in our department.

### Statistics

Subjects were grouped according to the percentage change in CI induced by VE after the LRM. Fluid responder patients (FR) were defined by an increase in CI ≥ 15% [10]. Four variations of PP and CVP during the LRM were analysed between the baseline level (peak inspiratory pressure 20 cmH_2_O, 5 cmH_2_O PEEP) and at four steps of the LRM: 30, 35, 40, and 45 cm H_2_0 of peak inspiratory pressure (Supplementary Figure S1). To assess the ability of ΔPP and ΔCVP parameters at each step of the LRM to identify fluid responsiveness, receiver-operating characteristic (ROC) curves were generated. The optimal threshold value (Youden’s index that maximizes the sum of the sensitivity and specificity) was determined. The areas under the ROC curves were calculated for each variable at the four steps of the LRM. We defined the grey zone, wherein a fluid responsiveness determination could not be made, for values with a sensitivity lower than 90% or specificity lower than 90% [11]. The method described by DeLong et al. was used to compare the areas under the ROC curve associated with the variables. Finally, we calculated the sensitivity and specificity of the combination of ΔPP and ΔCVP by plotting individual ΔPP values according to the corresponding ΔCVP value (Fig. 2) for the lowest (30 cm H_2_O peak inspiratory pressure) and the highest LRM stage (45 cm H_2_O). We defined the grey zone to be when the combination of ΔPP and ΔCVP had opposite value. A *p*-value < 0.05 was considered statistically significant. Statistical analyses were performed with XLSTAT software (version 2019.3.2) and Medcalc version 19.6.4.

## Results

Demographic characteristics and reasons for ICU hospitalization have been previously published [9]. Briefly, 9 patients (50%) were fluid responders (FR) and 9 were not (NFR). Area under the ROC curve analysis, sensitivity and specificity, and cut-off values with the number of patients in the grey zone ΔPP and ΔCVP at each step of the LRM (30, 35, 40, and 45 cm H_2_0 of peak inspiratory pressure) are summarized in Table 1.

**Table 1:** Diagnostic performance of absolute variations ΔPP and ΔCVP to predict fluid responsiveness for 30, 35, 40 and 45 cmH_2_O peak inspiratory pressures during LRM

		**AUC**	**95% CI**	**Cut-off (mmHg)**	**Grey Zone (mmHg)**			**Sensitivity (%)**	**Specificity (%)**
					Lower	Upper	patients (%)		
**Pinsp 45**	ΔPP	0.920	0.693 to 0.995	21	21.9	27.1	28	100	78
	ΔCVP	0.883	0.645 to 0.984	8	5.45	+ .1	33	78	89
**Pinsp 40**	ΔPP	0.895	0.661 to 0.988	12	14.7	24.2	44	100	67
	ΔCVP	0.833	0.586 to 0.964	6	3.9	7.1	44	78	89
**Pinsp 35**	ΔPP	0.914	0.685 to 0.993	7	8.8	19.1	39	100	78
	ΔCVP	0.901	0.669 to 0.990	4	2.9	4.1	39	78	89
**Pinsp 30**	ΔPP	0.951	0.736 to 0.999	4	3.9	6.4	28	89	89
	ΔCVP	0.887	0.637 to 0.981	2	1.9	3.1	50	89	67

**Fig. 1:**
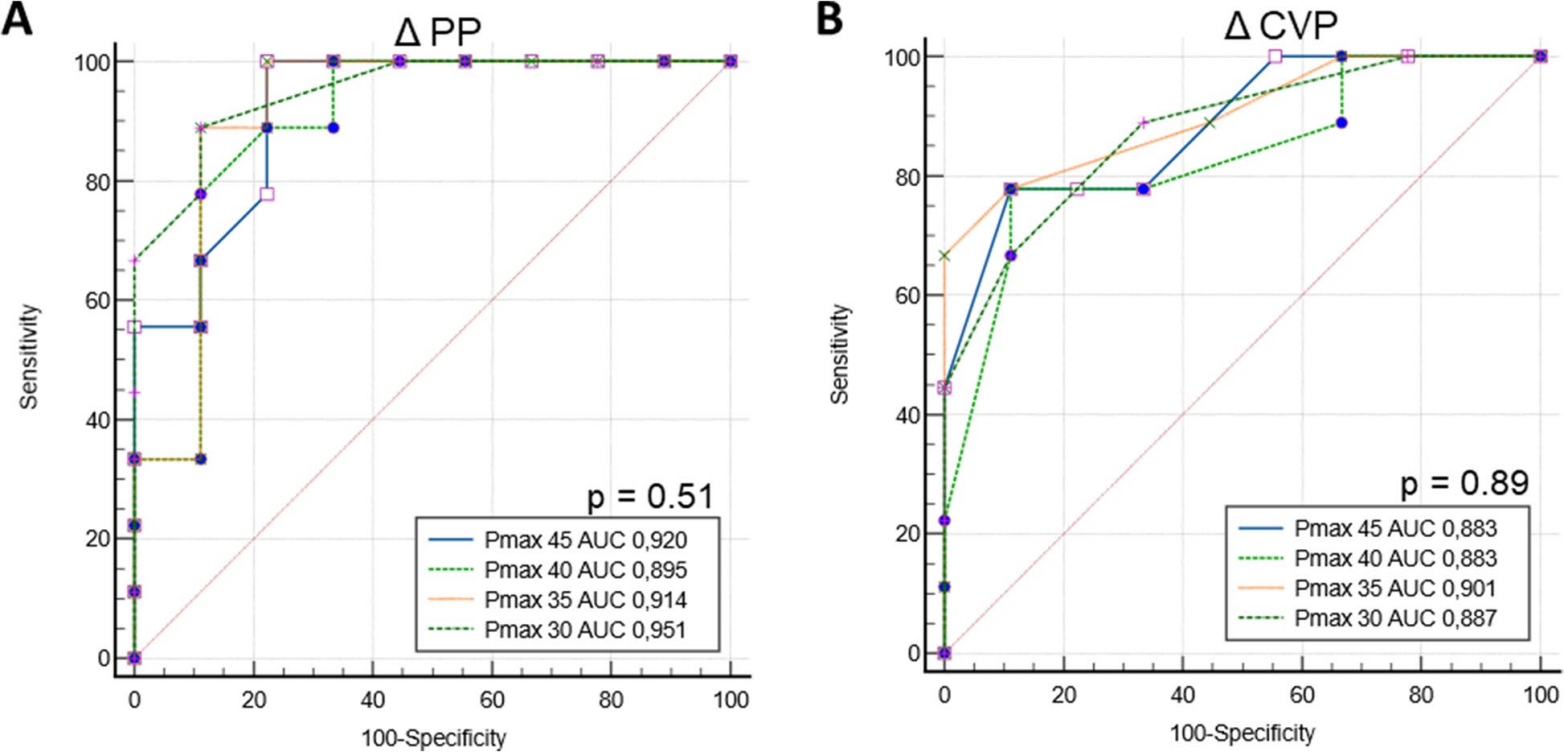
ROC curves comparing the ability of ΔPP (**A**) and ΔCVP (**B**) to discriminate between fluid responders and fluid non-responders for different peak airway pressures (Pinsp). Notes: *p*-value (P) is the statistical difference between ROC curves areas for Pinsp 30 cmH_2_O and Pinsp 45 cmH_2_O. Legends: AUC: Area Under the ROC Curve, Pinsp: step inspiratory pressure

ROC Curves comparing the ability of ΔPP and ΔCVP at each step of the LRM to discriminate FR from NFR are presented in Fig. 1.

**Fig. 2:**
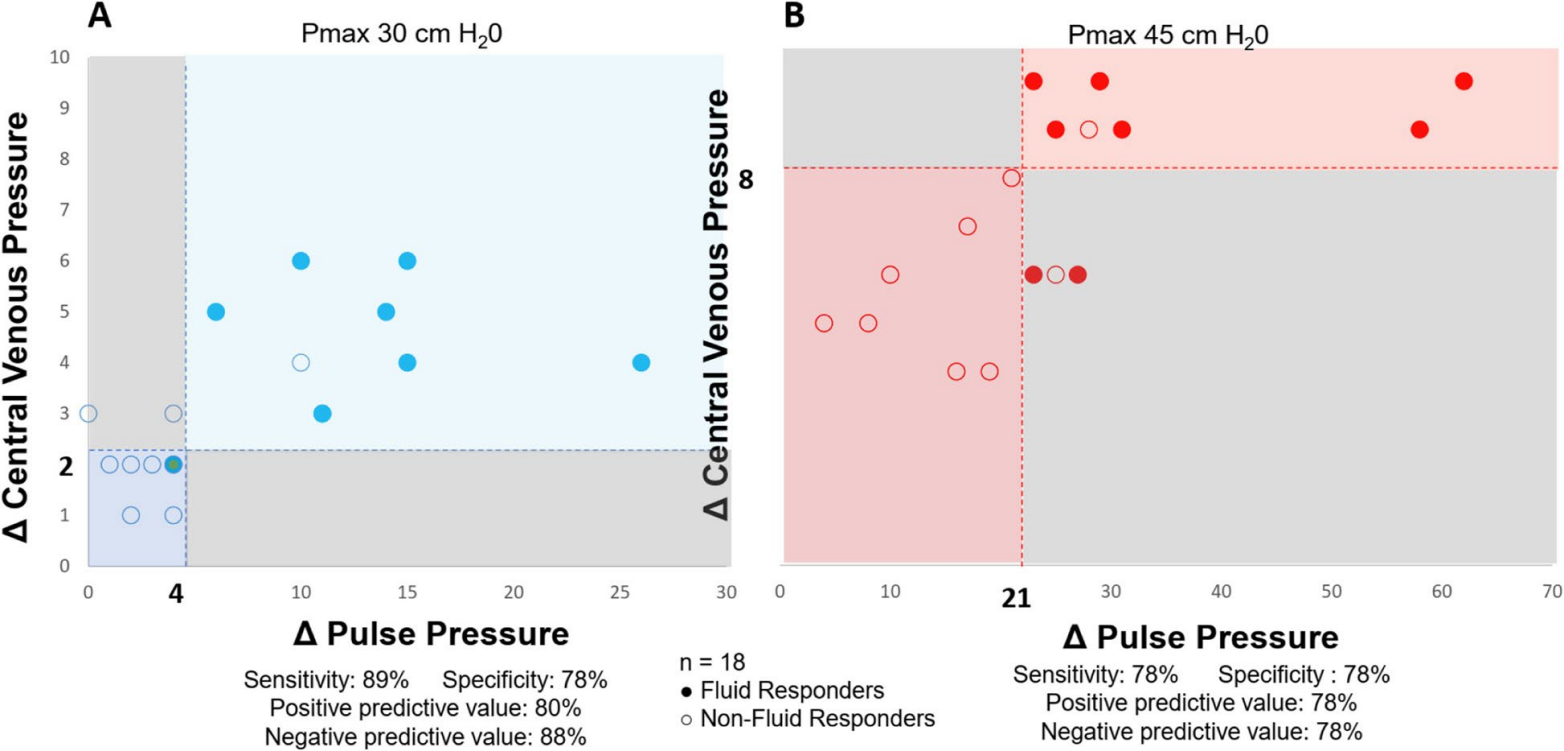
Individual data for ΔCVP and ΔPP during the LRM according to a 30 cmH_2_O (**A**) and a 45 cmH_2_O (**B**) peak airway pressure (**B**). Notes: Fluid responder patients are represented by closed circles and non-fluid responders by open circles. Three patients lay in the grey zone for the 45 cm H_2_0 peak airway pressure step versus two for the 30 cm H_2_0 peak airway pressure step. Sensitivity, specificity, positive and negative predictive values were calculated, and the test considered to be positive if values were both positive (upper right quadrant) or both negative (lower left quadrant)

AUC of ΔPP and ΔCVP at the lower step pressure of the LRM was 0.92 (95% CI 0.74 to 1.00) and 0.88 (95% CI 0.64 to 0.98), respectively. Comparison of ROC curves between the extreme steps of the LRM (peak airway pressure 30 versus 45 cmH_2_O) showed no significant difference for ΔPP (*p* = 0.51), and for ΔCVP (*p* = 0.89), respectively. At the lowest step pressure, combining both parameters left 11% of patients (2 out of 18) in the grey zone, versus 28% for ΔPP alone and 50% for ΔCVP alone. Negative and positive predictive values were 88% and 80% respectively, sensitivity and specificity were 89 and 78%, respectively (Fig. 2A) when both values were combined. During the highest step pressure of the LRM (45 cmH_2_0), the combination of ΔPP and ΔCVP left 17% of patients (3 out of 18) in the grey zone (Fig. 2B), with a sensitivity and a specificity of 78%.

## Discussion

This retrospective analysis of the original STEP-PEEP study [8] showed that absolute variations of pulse pressure and central venous pressure could predict fluid responsiveness in mechanically ventilated patients for a lower step pressure during a LRM (i.e. 30 cmH_2_O versus 45 cm H_2_0). The absolute cut-off value during the lower step pressure was 4 mmHg for ΔPP with a sensitivity of 89% and a specificity of 89% and an AUC of 0.95 (95% CI 0.74 to 1.0). The cut-off value was 2 mmHg for ΔCVP with a sensitivity of 90% and a specificity of 67% and an AUC of 0.89 (95% CI 0.64 to 0.98). Comparison of areas under the ROC curves between the extreme LRM step pressures showed no significant difference for ΔPP, and for ΔCVP, respectively. Finally, by combining ΔPP and ΔCVP, we observed a decrease in the percentage of patients in the grey zone from 17 to 11%. By comparison this percentage is lower than other methods used to predict fluid responsiveness, such as stroke volume variation induced by LRM [12] or pulse pressure variation in the ICU [13], both of which leave more than 50% of patients in the grey zone.

This ancillary analysis was conceived after reviewers of the initial study raised several limitations. The first objection was the use of high peak pressure during the LRM, when compared with the usual 30 cm H_2_0 peak pressure used in the OR [6]. Indeed, using lower inspiratory pressure may reduce LRM respiratory and hemodynamic complications. By showing that the first step at 30 cm H_2_0 of the LRM allows precise discrimination of FR from NFR patients with only 11% of patients in the grey zone when combining ΔPP and ΔCVP, we now propose a less aggressive LRM in the ICU setting to optimize fluid status. The second objection was the use of an indirect parameter to predict fluid responsiveness, i.e. the calculated angle between the correlation line (calculated from the variations of the CVP and PP according to the airway pressure step) and the horizontal line. We confess that the bedside applicability of this angle calculation is poor. Conversely, measuring the absolute value of a parameter before and after a dynamic test is routine, for example when calculating a significant variation of cardiac output when testing fluid responsiveness. We therefore reanalysed absolute variations of CVP and PP between the baseline and each pressure step of the LRM, from 30 to 45 cm H_2_0. By showing that analysis of absolute variations at the lowest step is as effective as at the highest step, we can recommend the use of absolute variations of PP and CVP as an easy method to determine fluid responsiveness using a lower peak pressure and a more straightforward calculation. The good sensitivity and specificity of ΔCVP allows the use of this index when PP is not accurate, such as atrial fibrillation for which the rate in ICU ranges from 10 to 60% [14, 15].

Combining a right cardiac preload variable (CVP) and a left cardiac function parameter (PP) is advantageous in potentially limiting the impact of confounding factors such as diastolic and systolic right and left cardiac function as well as arterial compliance when analysing the preload dependency state. Combining two parameters such as CVP and the shock index (HR/SAP) has been used to improve the positive or negative predictive value to better discriminate non FR patients [16]. Interestingly, in our study, combining ΔPP and ΔCVP did not improve the negative and the positive predictive value of ∆PP (NPV and PPV of 89%), although it did decrease the proportion of patients in the grey zone.

In 2008, Marik included 24 studies in a meta-analysis to test the ability of baseline CVP before VE or the variation of CVP before and after VE to predict fluid responsiveness. The pooled area under the ROC curve was only 0.56 (95% CI, 0.51 to 0.61). CVP has therefore been abandoned as a marker of fluid responsiveness, but its absolute value is still proposed as a marker of fluid overload [17]. Indeed, from a physiological point of view, CVP results from the interaction between the right ventricular (RV) function and the venous return. Consequently, a single CVP value may involve numerous cardiac function and venous return states. Therefore, CVP changes may be the consequence of variations in cardiac function, venous return or both [18]. However, when the venous return is stressed by an intrathoracic positive pressure (ITP), the balance point of the relationship between venous return and the right ventricular function is altered, moving the point towards the steep portion of the curve, and therefore determining a new intersection between venous return and CO. In the condition of hypovolemia, the point tends to be to the left of the curve, and a small increase of ITP would produce a large increase of CVP. This dynamic physiological approach has been tested during the mechanical respiratory cycle, wherein Cherpanath showed that a mean CVP variation of 12% induced by the positive ventilation could predict FR with an AUC of 1 (95% CI 0.85–1.00) after cardiac surgery [3]. Westphal had even shown in 2006 that the tight amplitude of CVP between insufflation and exsufflation could predict FR with an AUC of 0.9 [19]. These two parameters are however difficult to calculate at bedside.

Systemic arterial pulse pressure (PP) depends on the stroke volume (SV) and on the arterial compliance. The SV component can also be stressed by a variation of the ITP. The increase in ITP decreases the venous return and increases the right afterload, ultimately decreasing the SV. The inverse is observed when ITP decreases. If the hemodynamic status of the patient is on the steep part of the Frank Starling curve (low preload zone) then there may be a significant variation of SV when ITP changes. In the ICU setting, the pulse pressure variation (PPV) concept is based on the comparison between a zero-end-expiratory pressure (ZEEP) and the application of a PEEP during the mechanical respiratory cycle [20]. This approach has been extensively studied to show that PPV induced by the mechanical respiratory cycle could predict fluid responsiveness [21]. PPV is currently displayed by most commercial ICU monitors, but numerous limitations of PPV [22] as well as the scope data filtration process unique to each device prevents their use during a transient increase of PEEP. By increasing the ITP, LRM is another way to stress the left SV and consequently the PP, and has therefore been used as a dynamic test to predict FR alongside the passive leg raise and the end-expiratory occlusion tests [22]. In the ICU, variation of PP following an elevation of PEEP from 10 cm H_2_O to 20 cm H_2_O during an end-expiratory pause can predict FR with a moderate AUC of 0.72 (95% CI 0.50–0.94) [23]. In the same way, a decrease of 35% of PP induced by a peak pressure of 35 cm H20 during four seconds can predict FR with an AUC of 0.91 (CI95% 0.82–0.99) and a sensitivity of 75% and a specificity of 92% [24].

Compared to a peak pressure of 45 cm H_2_0, we did not find a better specificity for ΔCVP or a better sensitivity for ΔPP at a peak inspiratory pressure of 30cmH_2_0. Calculation of sensitivity and specificity depends on the chosen cut off value, and bedside application should depend on the clinical scenario. If the clinician feels that the potential risk associated with VE is low, a lower range of the grey zone should be chosen favoring high sensitivity. However if the clinician feels that the risk associated with VE is high (i.e. may lead to volume overload), then the upper range of the grey zone should be chosen, favoring high specificity.

A major strength of this study is the mode of data recording, wherein a video of the entire hemodynamic monitoring was recorded during the LRM, with the clinician announcing the time and pressure level for each step; thus, allowing for objective data collection and limiting bias introduced by the scope data filtration process, which is unique to each brand. Our data were later collected by pausing the video during the two last seconds of each LRM step, therefore ensuring no data loss.

This study has several limitations. As mentioned in the original study, norepinephrine infusion during this observational study may have impacted cardiac preload and reduced preload dependency [25]. However, the observed 50% fluid-responder rate is consistent with the existing literature [13]. The original study conducted a standardized LRM with increasing then decreasing pressure levels (Fig. 1). Our analysis and conclusions on fluid responsiveness prediction focused on the STEP-UP phase with increasing levels of pressure from 20 to 45 cmH_2_O. The STEP-DOWN phase, by prolonging the delay between the STEP-UP phase and the VE may have biased the result of the fluid responsiveness [26]. Future studies should compare the performance of a LRM at 30 cmH_2_O versus a LRM up to 45 cmH_2_O without the STEP-DOWN phase to verify our results. Moreover, our data comes from a small sample of patients. Extrapolation is therefore limited, and we emphasize that this is a pilot study. Finally, only data for pulse pressure and central venous pressure were tested in our study as they proved to be the two best parameters in the previous study.

## Conclusions

A step-up lung recruitment maneuver with a peak inspiratory pressure of 30 cm H_2_0 seems to give similar results for fluid responsiveness prediction as the previous linear interpolation model proposed in the STEP-PEEP study. Variations of central venous pressure and pulse pressure provide respectively good specificity and sensitivity for fluid responsiveness prediction and could be easily applicable at the bedside after validation by a larger prospective cohort study.

## Supplementary Information

**Additional file 1.**

## Data Availability

The datasets used and/or analysed during the current study are available from the corresponding author on reasonable request.
