# Supplementary material for: Variations of pulse pressure and central venous pressure may predict fluid responsiveness in mechanically ventilated patients during lung recruitment manoeuvre: an ancillary study
Source: BMC Anesthesiol. 2022 Aug 23;22:269. doi: 10.1186/s12871-022-01815-1 (PMC9396758; doi:10.1186/s12871-022-01815-1)
Supplement: Supplementary file 1 — Additional file 1. [file 12871_2022_1815_MOESM1_ESM.docx]

Supplementary Figure S1: Study design

STEP-PEEP lung recruitment maneuver

PP and CVP variations (Δ) were calculated at each step of inspiratory pressure (Pinsp). The STEP-DOWN phase was not considered.

Pinsp: step peak inspiratory pressure

Baseline: reference pressure level (Pinsp 20 cmH_2_0/PEEP 5 cm H_2_0)
